# DISPARITIES IN CONTINUITY OF CARE AND USE OF ANNUAL WELLNESS VISITS AMONG OLDER AMERICANS WITH DEMENTIA

**DOI:** 10.1093/geroni/igad104.0695

**Published:** 2023-12-21

**Authors:** Elham Mahmoudi

**Affiliations:** University of Michigan Medical School, Ann Arbor, Michigan, United States

## Abstract

Working on a risk-adjusted, prospective, capitated payment system, Medicare Advantage (MA) plans are incentivized to offer more efficient and coordinated care than traditional Medicare (TM). This study has two aims: (1) compare the continuity of care and use of annual wellness visits between MA and TM enrollees with Alzheimer’s disease and related dementia (ADRD), and (2) examine Hispanic-White and Black-White disparities in the continuity of care and annual wellness visits by comparing MA with TM enrollees with ADRD. We used a 20% random sample of TM and MA insurance claims (2018-2019). Participants included individuals 65+, with a diagnosis of ADRD, and two years of continuous enrollment in TM (n=129,177) or MA (n=119,130). We used the Bice-Boxerman Continuity of Care Index to measure continuity of care. Generalized linear models were applied. TM lowered the odds of Black and Hispanic individuals having an annual wellness visit [OR=.80 (95% CI:.79-.82)]. Black and Hispanic TM enrollees had lower odds of annual wellness visits as compared to their Black and Hispanic MA counterparts [OR=.83 (95% CI:78-.88) and OR=.88 (95% CI:.83-.94)], respectively. White, Black, and Hispanic MA enrollees had higher continuity of care than their counterparts in TM (27.4 vs. 24.9; 28.9 vs. 28.1; 32.1 vs. 30.1, respectively). MA enrollees with ADRD had higher care continuity than their TM counterparts. Compared to TM, MA increased the odds of annual wellness visits and reduced Hispanic-White disparity in annual wellness visits. Findings from this study can inform policies promoting preventive and equitable care for patients with ADRD.

